# Surgery.—Hernia

**Published:** 1904-06-04

**Authors:** 


### SURGERY.—HERNIA.

**The Congenital Origin of Hernia.**—Hamilton Russell[1] contributes this paper. He first refers to the following conclusions he arrived at in 1899:— 1. Oblique inguinal hernia is invariably caused by the presence of a congenital sac, which in the vast majority of cases is provided by patency of the whole or a portion of the processus vaginalis. 2. There is no evidence in favour of the view that congenital weakness of the abdominal wall in the inguinal region is a factor in the causation of inguinal hernia. 3. While actual weakness of the abdominal wall in the inguinal region is frequently met with, such weakness is not congenital, but is an acquired weakness due to the existence of the hernia during a lengthened period. 4. Complete removal of the sac, when performed before the abdominal wall has sustained such damage, will not be followed by recurrence. 5. It follows from the foregoing that the use of a truss (except in the case of a very young infant) is an improper method of treatment; the only permissible course to advocate for every case of hernia in a child is operative removal of the sac—the sole cause of the hernia. In 1902 the writer was able to produce a great weight of evidence in favour of the congenital origin of femoral hernia, and thought we might gain some information about direct inguinal hernia which would enable it to be placed in the same category. He describes a specimen bearing out this view. Continuing his evidence in favour of the congenital origin of femoral hernia, he points out the importance of sacculation in the sac, and he thinks that during early embryonic life, when the bud-like projections take place to form the lower extremities, a pouch of peritoneum may be included in the bud which includes the whole thickness of the body wall. This pouch may occur in more than one situation in the limb, but its most frequent seat is in the immediate vicinity of the main vessels and is the future crural canal. The writer further points out that there is a definite relationship between the three superficial branches of the femoral artery which run upwards on to the abdominal wall and the positions occupied by femoral sacs. The sacculated condition of the sac is assumed to be due to the tortuosities of the vessels which it follows. He formulates the following law, viz.: "The variations observed in the clinical manifestations of hernia are mainly determined by the size and position of the sac." The following classification of hernias is suggested :—

| | | |
|---|---|---|
| Congenital inguinal hernia | Funicular | Simple—total and partial funicular hernia. |
| | | Sacculated—interstitial, properitoneal, and infantile. |
| | Non-funicular direct inguinal hernia. | |
| Acquired inguinal hernia | Probably not existent (? direct hernia *sometimes*). | |

The larger sacculations and secondary sacs included under the term infantile hernia are attributed to the funicular process being caught in its descent by the abdominal wall and its further descent on either side of the point caught occurring in two sacs. The paper terminates with a discussion on the present inaccurate classification of hernias.

**A Case of Properitoneal Hernia.**—Betham Robinson[2] reports this case. A man aged 54 was admitted into St. Thomas's Hospital in January 1901 with symptoms of intestinal obstruction. He had had a left inguinal hernia for many years, which had been kept up by a truss. On admission, the abdomen was greatly distended, but the walls fairly flaccid. Nothing abnormal could be detected in the rectum, and both inguinal canals were free of any hernial tumour. The vomit was dark bile-stained, but not particularly offensive. Under chloroform, palpation detected some increased resistance on deep pressure just above the pubes on the left side. On opening the abdomen in the middle line a loop of small gut and matted omentum were found entering through a small opening into a pouch situated behind the left abdominal wall to the side of the bladder. After incising the edge of this opening the omentum could be withdrawn, but not the bowel, which seemed firmly adherent in front over the situation of the internal ring. The inguinal canal was then opened up from the front and disclosed an empty peritoneal sac, and on following this up to the internal ring there was seen presenting in, but not through, the opening adherent black bowel. It was then evident that the bowel occupied and was strangulated in a pouch behind the abdominal wall. The bowel was removed from the sac, and a gangrenous portion, $4\frac{1}{2}$ inches long, was resected, and an end-to-end junction made. The man died the following day. The autopsy revealed the double sac, one in the inguinal canal and the other intra-pelvic alongside the bladder and extending into the iliac fossa to just beyond the external iliac vessels. The opening from the latter sac into the former was at the junction of its outer and middle thirds to the outside of the deep epigastric artery. The intrapelvic sac was situated outside the transversalis fascia in the sub-peritoneal connective tissue. Biloculated hernias in the inguinal region are divided into two groups, first those in which both loculi are in front of the transversalis fascia, and there are three varieties of these: (*a*) the hour-glass sac with constriction at the site of the external ring; (*b*) that variety in which one portion of the sac spreads upwards and outwards over the anterior surface of the external oblique, and (*c*) that in which a diverticulum extends upwards between the layers of the muscular wall; secondly, those in which the second or deep sac lies behind the transversalis fascia. There are two subdivisions of this group: (*a*) the properitoneal hernia of Kronlein and the (*b*) prevesical hernia. The paper concludes with a discussion as to the origin of this hernia.

**A Case of Right Duodenal Hernia**.—Selby[3] reports this case. A man of 40 was admitted to hospital with symptoms of acute intestinal obstruction. He had had previous attacks of a similar nature before. On opening the abdomen a large peritoneal sac was found occupying the right half of the abdomen. The cæcum was empty and contracted, and coils of distended and deeply congested small intestine were found outside the sac on the left of the abdomen. The orifice of the sac was to the right of the middle line, a little below the umbilicus, looking forwards and to the left and measuring about 2½ inches by 1 inch. The free anterior margin was thick and contained vessels. The edge of the opening into the sac was divided between two ligatures and a considerable length of small gut drawn out, a few coils were adherent to the upper part. The patient died 24 hours after operation, and at the necropsy the seat of the opening into the sac was on the posterior abdominal wall just below and to the right of the duodeno-jejunal flexure. The omentum was adherent to the outer surface of the sac above. The seat of constriction was in the small intestine 8 inches from the ilio-cæcal junction and presumably the whole of the small intestine except the lower ileum had been in the sac. The ascending colon was pushed to the right, and the cæcum dragged upwards so that the vermiform appendix appeared to spring from its anterior surface.

1 Lancet, March 12, 1904. 2 Brit. Med. Jour, March 12, 1904. 3 Ibid.

(*To be continued.*)

